# Supplementary material for: Photodynamic Inactivation Enhances Antibiotic Efficacy Without Affecting Drug Stability: Insights into Photosensitizer–Antibiotic Combination Therapies
Source: Int J Mol Sci. 2025 Nov 21;26(23):11267. doi: 10.3390/ijms262311267 (PMC12692489; doi:10.3390/ijms262311267)
Supplement: Supplementary file 1 [file ijms-26-11267-s001.zip › ijms-3965311-supplementary.pdf]

Supporting Information

Photodynamic Inactivation Enhances Antibiotic Efficacy Without Affecting Drug Stability: Insights into Photosensitizer–Antibiotic Combination Therapies

Rocío B. Acosta, Edgardo N. Durantini and Mariana B. Spesia *

IDAS-CONICET, Departamento de Química, Facultad de Ciencias Exactas, Físico-Químicas y Naturales, Universidad Nacional de Río Cuarto, Ruta Nacional 36 Km 601, Río Cuarto X5804BYA, Córdoba, Argentina; racosta@exa.unrc.edu.ar (R.B.A.); edurantini@exa.unrc.edu.ar (E.N.D.)

* Correspondence: mspesia@exa.unrc.edu.ar; Tel./Fax: +54-358-4676521

1. Supplemental Tables

Table S1. Viable count of *S. aureus* after 8 h of incubation at 37 °C

**Assay**	**log CFU/mL**
Control	9.80
Dark Control	9.73
$TMAP^{4+}$	9.37
RIF	9.58
AMP	9.33
$TMAP^{4+}$ + AMP	8.93
$TMAP^{4+}$ + RIF	8.85

Table S2. Viable count of *E. coli* after 8 h of incubation at 37 °C

**Assay**	**log CFU/mL**
Control	8.79
Dark Control	8.73
$TMAP^{4+}$	8.44
CFX	8.56
$TMAP^{4+}$ + CFX	8

2. Supplemental Figures

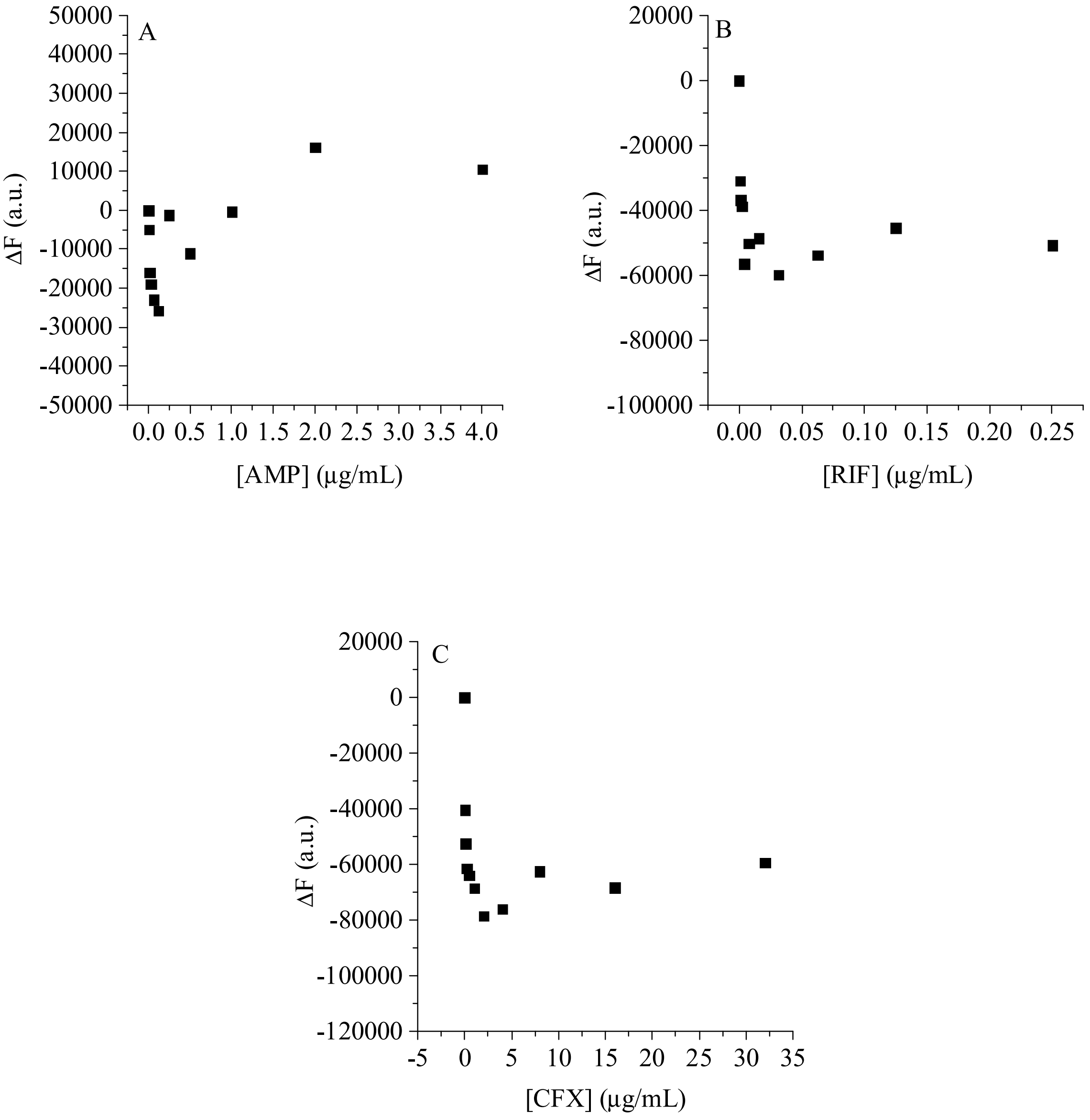

Figure S1. Plots of $\Delta F = (F - F_0)$ of $TMAP^{4+}$ (0.5 μM, λ_{exc}= 417 nm) *vs* [ATB] of (A) [AMP], (B) [RIF] and (C) [CFX] in water.

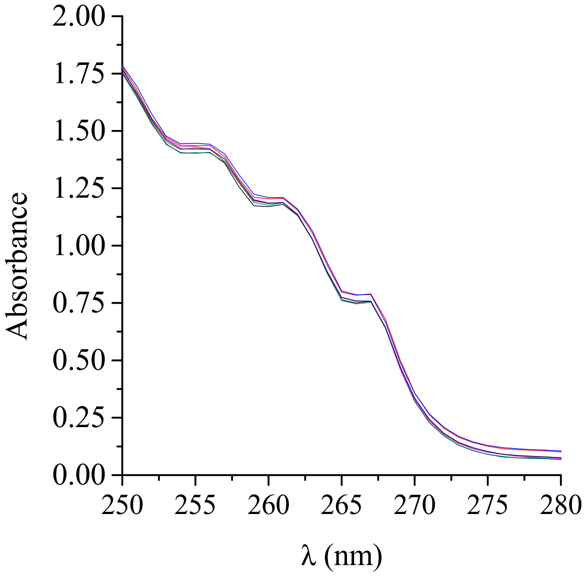

Figure S2. Variation of UV–vis absorption spectra of AMP (2 μg·mL^{-1}) after different irradiation times with an irradiance of 90 mW·cm^{-2} at room temperature in water.

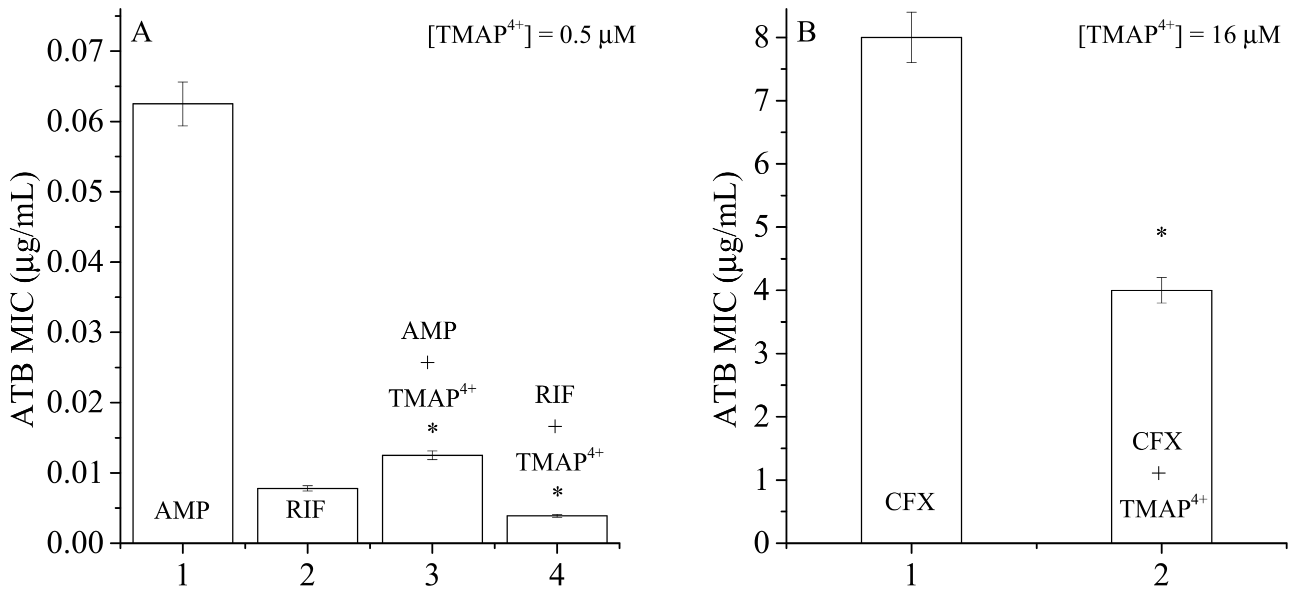

Figure S3. Comparison of MICs of ATBs alone and in combination with PDI at a fixed concentration of $TMAP^{4+}$ against (A) *S. aureus* and (B) *E. coli.* **p* < 0.05, ATB MIC compared with the treatment alone.

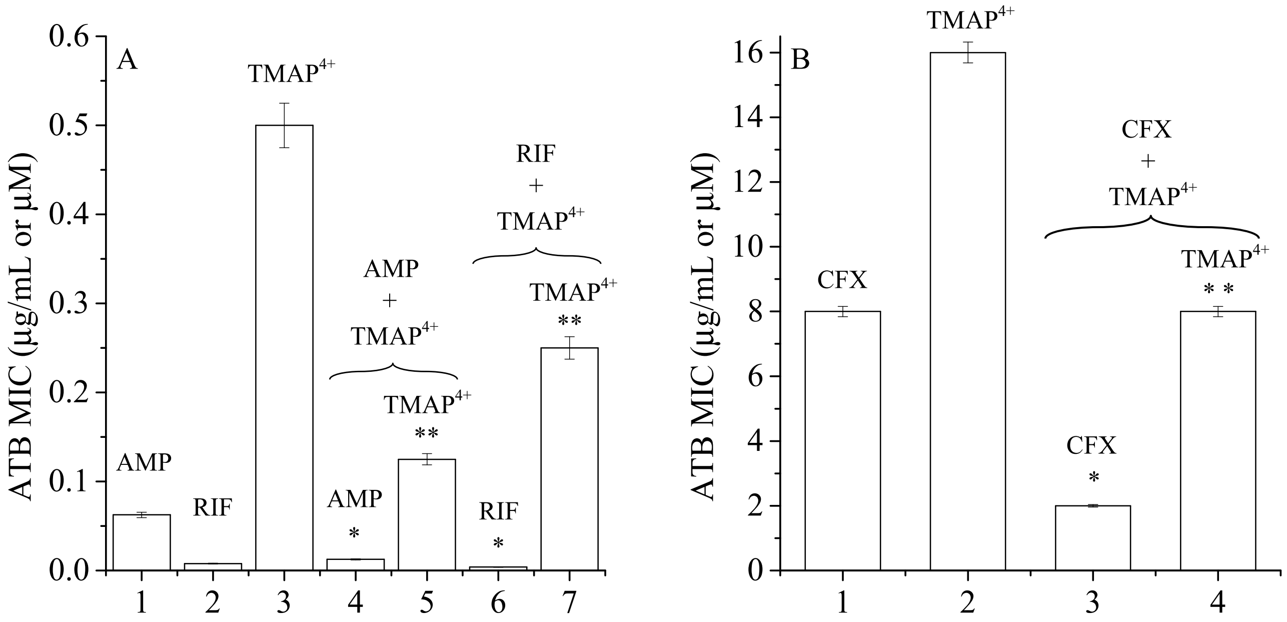

Figure S4. Comparison of MICs of ATBs alone and in combination with PDI at varying concentrations of both agents against (A) *S. aureus* and (B) *E. coli*. *$p < 0.05$, ATB MIC compared with the treatment alone. **$p < 0.05$, $TMAP^{4+}$ MIC compared with the treatment alone.
